# Hospitalisation for cirrhosis in Australia: disparities in presentation and outcomes for Indigenous Australians

**DOI:** 10.1186/s12939-020-1144-6

**Published:** 2020-02-17

**Authors:** Patricia C. Valery, Paul J. Clark, Gregory Pratt, Christina M. Bernardes, Gunter Hartel, Maree Toombs, Katharine M. Irvine, Elizabeth E. Powell

**Affiliations:** 1 QIMR Berghofer Medical Research Institute, 300 Herston Road, Herston, QLD 4006 Australia; 2 Centre for Liver Disease Research, Translational Research Institute, Faculty of Medicine, The University of Queensland, Brisbane, Australia; 3 Department of Gastroenterology and Hepatology, Mater Hospitals, Brisbane, QLD Australia; 4 Rural Clinical School, Faculty of Medicine, University of Queensland, Toowoomba, QLD Australia; 5 Mater Research, University of Queensland, Brisbane, QLD Australia; 6 Department of Gastroenterology and Hepatology, Princess Alexandra Hospital, Brisbane, QLD Australia

**Keywords:** Liver disease, Survival, Indigenous Australians, Epidemiology

## Abstract

**Background:**

Indigenous Australians experience greater health disadvantage and have a higher prevalence of many chronic health conditions. Liver diseases leading to cirrhosis are among the most common contributor to the mortality gap between Indigenous and other Australian adults. However, no comparative data exist assessing differences in presentation and patient outcomes between Indigenous and non-Indigenous Australians hospitalised with cirrhosis.

**Methods:**

Using data from the Hospital Admitted Patient Data Collection and the Death Registry, this retrospective, population-based, cohort study including all people hospitalised for cirrhosis in the state of Queensland during 2008–2017 examined rate of readmission (Poisson regression), cumulative survival (Kaplan–Meier), and assessed the differences in survival (Multivariable Cox regression) by Indigenous status. Predictor variables included demographic, health service characteristics and clinical data.

**Results:**

We studied 779 Indigenous and 10,642 non-Indigenous patients with cirrhosis. A higher proportion of Indigenous patients were younger than 50 years (346 [44%] vs. 2063 [19%] non-Indigenous patients), lived in most disadvantaged areas (395 [51%) vs. 2728 [26%]), had alcohol-related cirrhosis (547 [70%] vs. 5041 [47%]), had ascites (314 [40%] vs. 3555 [33%), and presented to hospital via the Emergency Department (510 [68%] vs. 4790 [47%]). Indigenous patients had 3.04 times the rate of non-cirrhosis readmissions (95%CI 2.98–3.10), 1.35 times the rate of cirrhosis-related readmissions (95%CI 1.29–1.41), and lower overall survival (17% vs. 27%; unadjusted hazard ratio (HR) = 1.16 95%CI 1.06–1.27), compared to non-Indigenous patients. Most of the survival deficit was explained by Emergency Department presentation (adj-HR = 1.03 95%CI 0.93–1.13), and alcohol-related aetiology (adj-HR = 1.08 95%CI 0.99–1.19). The remaining survival deficit was influenced by the other clinico-demographic and health service factors (final adj-HR = 1.08 95%CI 0.96–1.20).

**Conclusions:**

There was evidence of differential presentation, higher rates of readmissions, and poorer survival for Indigenous Australians with cirrhosis, compared to other Australians. The increased prevalence of Emergency Department presentation among Indigenous patients suggests missed opportunities for early intervention to prevent progressive cirrhosis complications and hospital readmissions.

## Introduction

Aboriginal and/or Torres Strait Islander Australians experience greater social and health disadvantage and have a higher prevalence of many chronic health conditions including liver disease [1, 2]. The complex interplay of socio-environmental factors (e.g. financial stress, food insecurity, substandard and overcrowded housing, remoteness of residence) [2] and racism [3], contribute to the high burden of chronic health conditions among Indigenous Australians. Ongoing barriers to health system access and use experienced by Indigenous Australians (e.g. mistrust of the health system, stigma, fear, a lack of cultural understanding) influence Indigenous patients’ engagement in the health system [4, 5]. Compared to non-Indigenous Australians, Indigenous Australians experience marked disparities in morbidity and mortality due to many chronic diseases. For example, cancer mortality and survival are largely attributed to being diagnosed later, receiving less treatment, and experiencing higher rates of comorbidities [6].

Cirrhosis disproportionately affect Indigenous Australians [7] and liver diseases are among the most common contributor to the mortality gap between Indigenous and other Australians aged 35–74 years [8]. Moreover, the only two studies examining hospital admission rates for cirrhosis among Indigenous Australians showed that they were overrepresented among patients admitted with cirrhosis [7, 9]. In 1995 in the state of Western Australia, Indigenous Australian men were 12.6 times more likely to be admitted to hospital with alcoholic cirrhosis than their non-Indigenous counterparts [9]. More recently, in a population-based study in Queensland, the rate of cirrhosis hospitalization for Indigenous Australians was 3.4 times that for non-Indigenous Queenslanders [7]. The reasons for the overrepresentation of Indigenous Australians among patients hospitalized with cirrhosis have not been examined.

Optimising care for patients with cirrhosis is complex, particularly decompensated cirrhosis, as these patients may be prescribed multiple medications, require dietary restrictions, and experience recurrent hospital admissions [10, 11]. The complexity of treatment has prompted the implementation of management programmes for cirrhosis [12–14]. Such programmes have, in general, a patient-centred approach to health care delivery with multidisciplinary, proactive, longitudinal quality care between visits, rather than episodic, symptom driven care. In particular, programmes that provide alternatives to hospitalization (e.g. “day-hospital” facility for invasive procedures) have shown reduction of hospital readmissions due to cirrhosis complications [12, 13]. Greater integration between primary care, community care organizations and liver specialists, with improved communication and information transfer is also seen as a way forward to improved patient outcomes [15].

In this article, we assessed differences in presentation and patient outcomes between Indigenous and non-Indigenous Australians hospitalised for cirrhosis. We aim to understand why hospitalization for cirrhosis is more frequent for Indigenous people in the state of Queensland [7], which constitutes 23% of Australia’s land mass.

## Methods

We conducted a retrospective, population-based, cohort study (longitudinal design) of people hospitalised for cirrhosis in Queensland during 2008–2017. Data were ascertained from the Queensland Hospital Admitted Patient Data Collection that contains information on hospital episodes of care for patients admitted to a Queensland hospital and the Queensland Death Registry. We identified all hospital admissions for cirrhosis in patients aged 20 years or older (*n* = 34,678 hospitalisation records). Admissions where the patient’s age or residential location was unknown and people whose primary residence was interstate or overseas were excluded.

Details regarding the selection of hospital admissions for cirrhosis have been described previously [7]. Briefly, all patients discharged from a Queensland hospital with a diagnosis of cirrhosis, or related complications, and/or had a procedure code related to cirrhosis or liver cancer, and/or died with a principal or other cause of death of cirrhosis, or related complications during 1/7/2007 to 31/12/2016 were ascertained. The study cohort was identified via a list of diagnosis and procedure codes provided to the Statistical Analysis Linkage Unit. An admission for cirrhosis was defined by hospitalization for a primary diagnosis of any of the following: alcoholic fibrosis and sclerosis of liver, alcoholic cirrhosis of liver, alcoholic hepatic failure, chronic hepatic failure, fibrosis and cirrhosis of liver, primary biliary cirrhosis/cholangitis, secondary biliary cirrhosis, biliary cirrhosis, unspecified, other and unspecified cirrhosis of liver, portal hypertension, hepatorenal syndrome, gastroesophageal varices, and hepatocellular carcinoma (HCC). To minimise the risk of missing cases, the definition also included hospitalization with any of the abovementioned diagnoses as “*other*” diagnosis and a cirrhosis-related diagnosis or procedure as primary diagnosis (e.g. abdominal paracentesis, endoscopic banding of oesophageal varices).

### Measurements

Demographic and health service characteristics and clinical data were obtained from Queensland Hospital Admitted Patient Data Collection. Date and cause of death were obtained from the Queensland Death Registry.

In Australia, a person is considered Indigenous if they are of Aboriginal and/or Torres Strait Islander descent, identify as Aboriginal and/or Torres Strait Islander, and are accepted as an Aboriginal or Torres Strait Islander person in the community in which they live, or have lived [16]. Indigenous status for an individual may vary across records for the same individual as Indigenous status is defined by self-assessment [17]. Using a validated algorithm for identification of Indigenous status from hospital linked data, patients were coded as Indigenous if at least one of their records within the study period identified them as Indigenous [17].

Patients’ residential postcodes were used to determine patients’ index of relative socioeconomic disadvantage score [18] and remoteness of residence [19]. Code lists for identification of cases, aetiology and cofactors were reviewed by four hepatologists and the Principal Statistical Data Quality Officer, Statistical Services Branch, Queensland Health [7]. Comorbidity was measured using the Charlson Comorbidity Index (Charlson index) [20] using validated coding algorithms [21].

We selected the first hospitalisation record for cirrhosis of each patient (referred to here as index admission) to avoid duplicate counting of individuals. When patients were transferred within the same or to another hospital, we considered this as one hospital stay. Length of stay was calculated by adding all days the patient was an admitted patient during one hospital stay.

### Data analysis

Analyses were conducted using Stata/SE (Version 15; Stata Corporation, College Station, TX). Numbers and percentages were prepared and the Chi-square test was used to compare groups. All *p* values were 2-sided. The rate of readmissions was calculated using person years at risk (PYAR) as a denominator (from index admission to 31 Dec 2017 or date of death, whichever came sooner). Poisson regression was undertaken to compare rate of readmission by Indigenous status. The vce (robust) option was used to obtain robust standard errors for the parameter estimates to control for mild violation of underlying assumptions. Incidence rate ratios (IRR) and 95% confidence interval (CI) were reported.

Cumulative overall survival estimates by Indigenous status were calculated using the Kaplan–Meier method (log-rank statistic). All cases were followed until date of death, or December 31, 2017, whichever came sooner. The survival time for patients who died on the index admission was counted as half a day. Multivariable Cox regression analysis reported in terms of hazard ratios (HRs) with associated 95% confidence intervals (CIs) was used to assess the differences by Indigenous status with respect to survival. Informed by our previous work [7], we included in the main effects model factors that could influence overall survival, such as patients’ sociodemographic features, disease aetiology, presence of comorbidity and complications of cirrhosis at index admission. We have excluded factors where prevalence of exposure in Indigenous patients was < 10%. In many patients with cryptogenic cirrhosis, non-alcoholic steatohepatitis (NASH) may be the primary cause of cirrhosis, as the diagnostic features of steatohepatitis may disappear as the liver disease progresses [22, 23]. We have therefore combined cryptogenic and non-alcoholic fatty liver disease (NAFLD)/NASH cirrhosis. We have also combined viral hepatitis B and C. When the overall model was statistically significant, a Least Absolute Shrinkage and Selection Operators (LASSO) penalised regression cox proportional hazards model was used to identify the set of variables that had the strongest association with the survival outcome. The LASSO procedure was used due to the high number of predictors (demographic, health service characteristics and clinical data) and potentially complex patterns of collinearity among predictor variables. If a set of variables are highly collinear, the LASSO only picks out one from the set, so it leads to a more parsimonious model. Variables included in the model were checked to ensure that they adhered to the assumption of proportional hazards over time (Shoenfeld residuals). The vce (robust) option was used to obtain robust standard errors for the parameter estimates to control for mild violation of underlying assumptions.

## Results

During 1 January 2008 and 31 December 2017, 11,448 people with cirrhosis aged 20 years or older were admitted to a hospital in Queensland: 779 (7%) were identified as Indigenous Australians, 10,642 (93%) as non-Indigenous Australians and for 27 (< 1%) Indigenous status was not available. Therefore, data for 11,421 patients were included in our study.

### Demographic and clinical features

Indigenous patients were significantly younger (346 (44%) had < 50 years vs. 2063 [19%]), and 480 (67%) had no partner (vs. 4880 [48%]) when compared to non-Indigenous patients; Table 1). Three hundred ninety-five Indigenous patients (51%) lived in most disadvantaged areas, and 154 (20%) lived in remote/very remote areas compared 25 and 1% of 10,642 non-Indigenous patients, respectively.

**Table 1:** Patient sociodemographic and health service characteristics at index hospital admission by Indigenous status

		Indigenous	Non-Indigenous	Total	*p*-value
		*N* = 779 (%)	*N* = 10,642 (%)	*N* = 11,421 (%)	
Age group (years) ^a^	20–29 years	12 (2%)	142 (1%)	154 (1%)	< 0.001
	30–39 years	100 (13%)	469 (4%)	569 (5%)	
	40–49 years	234 (30%)	1452 (14%)	1686 (15%)	
	50–59 years	261 (34%)	3215 (30%)	3476 (30%)	
	60–69 years	124 (16%)	2948 (28%)	3072 (27%)	
	70 years and over	48 (6%)	2416 (23%)	2464 (22%)	
Gender	Male	506 (65%)	7237 (68%)	7743 (68%)	0.08
	Female	273 (35%)	3405 (32%)	3678 (32%)	
Marital status^b^	Married/De facto	236 (33%)	5230 (52%)	5466 (51%)	< 0.001
	No partner	480 (67%)	4880 (48%)	5360 (50%)	
Rurality of residence	Major city	247 (32%)	6577 (62%)	6824 (60%)	< 0.001
	Inner regional	152 (20%)	2392 (23%)	2544 (22%)	
	Outer regional	226 (29%)	1544 (15%)	1770 (16%)	
	Remote/very remote	154 (20%)	129 (1%)	283 (3%)	
Socioeconomic advantageand and disadvantage	Q1 most affluent	44 (6%)	1596 (15%)	1640 (14%)	< 0.001
	Q2	85 (11%)	1874 (18%)	1959 (17%)	
	Q3	93 (12%)	2131 (20%)	2224 (20%)	
	Q4	162 (21%)	2313 (22%)	2475 (22%)	
	Q5 most disadvantaged	395 (51%)	2728 (26%)	3123 (27%)	
Hospital sector	Public hospital only	737 (95%)	7693 (72%)	8430 (74%)	< 0.001
	Private hospital only or mix	42 (5%)	2949 (28%)	2991 (26%)	
Health insurance status	Hospital Insurance	43 (6%)	3408 (33%)	3451 (31%)	< 0.001
	Not insured	720 (94%)	7036 (67%)	7756 (69%)	

^a^Data available by 5-year age groups, capped at 75+ years

^b^Marital status missing for 595 admissions

### Aetiology

While the most commonly recorded aetiology was alcohol for both groups, 547 (70%) Indigenous patients had alcohol-related cirrhosis compared to 5041 non-Indigenous patients (47%; Table 2). Notably, 48 (6%) of Indigenous patients had Hepatitis B virus (HBV) related cirrhosis (vs. 459 [4%]), and 23 Indigenous patients (3.0%) had NAFLD/NASH (vs. 560 [5%] and 172 cryptogenic (22% vs. 3199 [30%]) cirrhosis compared to their non-Indigenous counterparts.

**Table 2:** Diagnosis, comorbidities, cirrhosis-related complications, and health service factors at index admission by Indigenous status

	Indigenous	Non-Indigenous	Total	*p*-value
	*N* = 779 (%)	*N* = 10,642 (%)	N = 11,421 (%)	
Presumed aetiology				
Alcohol	547 (70%)	5041 (47%)	5588 (49%)	< 0.001
Cryptogenic or unspecified cirrhosis	172 (22%)	3199 (30%)	3371 (30%)	< 0.001
Chronic HCV	168 (22%)	1999 (19%)	2167 (19%)	0.06
NAFLD or NASH	23 (3%)	560 (5%)	583 (5%)	0.005
Chronic HBV	48 (6%)	459 (4%)	507 (4%)	0.02
Comorbidities				
Charlson comorbidity index^a^				
0 (no comorbidity)	446 (57%)	6188 (58%)	6634 (58%)	0.04
1	99 (13%)	1165 (11%)	1264 (11%)	
2	109 (14%)	1804 (17%)	1913 (17%)	
≥ 3	125 (16%)	1485 (14%)	1610 (14%)	
Diabetes	192 (25%)	2080 (20%)	2272 (20%)	< 0.001
Cancer	95 (12%)	1840 (17%)	1935 (17%)	< 0.001
Moderate or severe renal disease	76 (10%)	631 (6%)	707 (6%)	< 0.001
Congestive heart failure	63 (8%)	523 (5%)	586 (5%)	< 0.001
Drug use/abuse as cofactor	29 (4%)	243 (2%)	272 (2%)	0.01
Complications of cirrhosis				
Ascites	314 (40%)	3555 (33%)	3869 (34%)	< 0.001
Gastrointestinal bleeding	248 (32%)	4055 (38%)	4303 (38%)	< 0.001
Hepatocellular carcinoma	79 (10%)	1396 (13%)	1475 (13%)	0.02
Hepatic encephalopathy	52 (7%)	507 (5%)	559 (5%)	0.02
Jaundice	12 (2%)	87 (1%)	99 (1%)	0.04
Hepatorenal syndrome	24 (3%)	335 (3%)	359 (3%)	0.92
Spontaneous acute peritonitis	36 (5%)	310 (3%)	346 (3%)	0.01
Referral source				
Emergency department	510 (68%)	4790 (47%)	5300 (48%)	< 0.001
Outpatient clinic from this/other hospital	133 (18%)	2484 (24%)	2617 (24%)	
Private medical practitioner	37 (5%)	2274 (22%)	2311 (21%)	
Other	73 (10%)	724 (7%)	797 (7%)	
Length of hospital stay				
1 day	108 (14%)	3056 (29%)	3164 (28%)	< 0.001
2–4 days	186 (24%)	2071 (20%)	2257 (20%)	
5–9 days	218 (28%)	2323 (22%)	2541 (22%)	
10–19 days	156 (20%)	1763 (17%)	1919 (17%)	
20–29 days	47 (6%)	617 (6%)	664 (6%)	
30+ days	64 (8%)	812 (8%)	876 (8%)	
Utilised allied health service				
Pharmacist	108 (14%)	1776 (17%)	1884 (17%)	0.04
Dietician	292 (38%)	3500 (33%)	3792 (33%)	0.02
Social worker	208 (27%)	2425 (23%)	2633 (23%)	0.01
Occupational therapist	104 (13%)	1759 (17%)	1863 (16%)	0.02
Physiotherapist	223 (29%)	3074 (29%)	3297 (29%)	0.88
Palliative care	26 (3%)	447 (4%)	473 (4%)	0.24

^a^Higher scores indicate higher comorbidity burden

### Cirrhosis complications

Ascites was the most frequent complication of cirrhosis (34% overall). 314 (40%) Indigenous patients had ascites, 52 (7%) hepatic encephalopathy, and 12 (2%) jaundice, compared to 33, 5, 1% of 10,642 non-Indigenous patients. 4055 (38%) non-Indigenous patients had gastrointestinal bleeding and 1396 (13%) had HCC, compared to 248 (32%) and 79 (10%) Indigenous patients, respectively. A higher proportion of Indigenous patients were admitted as unplanned or Emergency Department presentations compared to non-Indigenous (510 [68%] vs. 4790 [47%], respectively; *p* < 0.001).

### Comorbidities

Indigenous patients had significantly higher Charlson index scores than non-Indigenous patients (*p* = 0.04). Compared to non-Indigenous patients, Indigenous patients had higher prevalence of diabetes (192 [25%] vs. 2080 [20%]; *p* < 0.001), chronic renal disease (76 [10%] vs. 631 [6%]; p < 0.001) and congestive heart failure (63 [8%] vs. 523 [5%]; p < 0.001), and lower prevalence of cancer (95 [12%] vs. 1840 [17%]; p < 0.001). Indigenous and non-Indigenous patients were similar with regard to the other diseases listed in the Charlson index. As drug use/abuse is a cofactor for HBV and HCV-related cirrhosis, we have examined these. 29 (4%) Indigenous patients had drug use/abuse listed as co-factors compared to 243 (2%) non-Indigenous patients (*p* = 0.01).

### Burden of care

Length of hospital stay varied significantly by Indigenous status, with fewer Indigenous patients having one-day admissions (108 [14%] vs. 3056 [29%]; *p* < 0.001). The median length of hospital stay was 7 days (interquartile range (IQR) 3–13) compared to 5 days (IQR 1–11) for their non-Indigenous counterparts. As an inpatient, Indigenous patients frequently accessed multidisciplinary health services (dietician, physiotherapist, social worker, pharmacist and occupational therapist). A higher proportion of Indigenous patients utilised the service of a dietitian (292 [38%] vs. 3500 [33%]; *p* = 0.01) and social worker (208 [27%] vs. 2425 [23%]; p = 0.01), and a higher proportion of non-Indigenous were assessed by a pharmacist (108 Indigenous [14%] vs. 1,776 non-Indigenous [17%]; *p* = 0.04) and occupational therapist (104 [13%] vs. 1759 [17%]; *p* = 0.02). Only 4% of patients utilised hospital-based palliative care services, with no difference by Indigenous status (*p* = 0.24).

Once discharged, Indigenous patients with cirrhosis were more likely to be readmitted than non-Indigenous patients, whether for liver disease or other reasons. Indigenous Australians had 3 times the rate of non-cirrhosis-related admissions compared to non-Indigenous patients (IRR = 3.04 95%CI 2.98–3.10) and a higher rate of cirrhosis-related admissions (IRR = 1.35 95%CI 1.29–1.41). Overall, the rate of readmissions was 86.3 admissions/10,000 PYAR, 21.1/10,000 PYAR for cirrhosis-related readmissions and 65.2/10,000 PYAR for admissions not related to cirrhosis (Table 3).

**Table 3:** Rate of re-admissions per 10,000 person years at risk by Indigenous status

	All re-admissions			Cirrhosis-related re-admissions			Non-cirrhosis-related re-admissions		
	Readmissions N	Rate per 10,000 PYAR	IRR (95%CI)	Readmissions N	Rate per 10,000 PYAR	IRR (95%CI)	Readmissions N	Rate per 10,000 PYAR	IRR (95%CI)
Overall	95,865	86.3	–	23,413	21.1	–	72,452	65.2	–
Indigenous	13,714	204.5	2.60 (2.55–2.65)	1866	27.8	1.35 (1.29–1.41)	11,848	176.6	3.04 (2.98–3.10)
Non-Indigenous	82,151	78.7	1.00	21,547	20.6	1.00	60,604	58.1	1.00

### Survival

At the end of the follow-up period 61% of Indigenous patients had died compared to 57% of non-Indigenous patients (*p* = 0.03). A higher proportion of Indigenous patients died in hospital, rather than not (e.g. died at home, at a hospice), compared to non-Indigenous patients (74% vs. 70%, *p* = 0.05).

The major cause of death in both groups was cirrhosis or cirrhosis-related complications (4208 [65%]; *p* = 0.36 for the difference between groups). Among Indigenous patients, the most frequent cause of death was alcohol-related cirrhosis (132 [28%]), followed by deaths due to cirrhosis complications (66 [14%], the majority of which (49 out of 66 [88%]) were HCC). Among non-Indigenous patients, the most frequent cause of death was cirrhosis-related complications (1416 [23%], the majority of which (1126 out of 1416 [89%]) were HCC), followed by deaths due to alcohol-related cirrhosis (1206 [20%]).

**Fig. 1:**
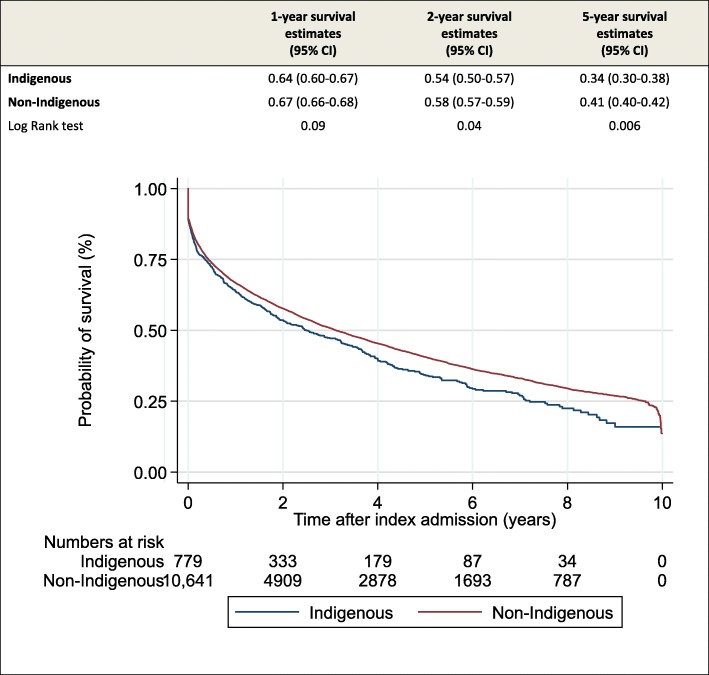
Relative survival at 1, 2 and 5 years after index admission and cumulative survival (estimated by Kaplan Meier method) by Indigenous status

The median time from index admission to death was 1.68 years (IQR 0.32–4.25) overall, 1.47 years (IQR 0.24–3.81) for Indigenous patients with cirrhosis and 1.70 years (IQR 0.33–4.29) for non-Indigenous patients. Across 2- and 5-year survival estimates, Indigenous patients have a significantly lower survival compared to non-Indigenous patients (Fig. 1). The probability of overall survival was 17% (95%CI 12.5–22.6) and 27% (95%CI 25.6–28.2), respectively (*p* = 0.002). These disparities were reflected in the unadjusted hazard rate, which was 16% higher for Indigenous patients compared to their non-Indigenous counterparts (HR = 1.16 95%CI 1.06–1.27).

In univariable analysis of overall survival, all factors examined were significantly associated with survival (Table 4). Age, variation in referral source (emergency presentations), and alcohol-related cirrhosis had the strongest influence in the adjusted difference in survival between the two groups. In multivariable analysis, adjusting for the younger age of Indigenous patients increased the HR to 1.30 (95%CI 1.18–1.42). Adding referral source (adj-HR = 1.03 95%CI 0.93–1.13), and alcohol-related cirrhosis (adj-HR = 1.08 95%CI 0.99–1.19), one at a time, explained most of the survival deficit. Addition of other variables (demographic and clinical factors, and health service use), one at a time, led to slight changes in the hazard ratio for Indigenous status (data not shown). The final (full model) adjusted hazard ratio was 1.08 (95%CI 0.96–1.20). There were 13 independent factors significantly associated with survival. The highest adjusted HRs were for having cancer (adj-HR = 2.59 95%CI 2.26–2.97), followed by age ≥ 50 years (adj-HR = 1.57 95%CI 1.45–1.69), emergency presentation (adj-HR = 1.56 95%CI 1.46–1.67), ascites (adj-HR = 1.47 95%CI 1.37–1.57), and Charlson index> 0 (adj-HR = 1.46 95%CI 1.36–1.56).

**Table 4:** Predictors of mortality among 11,421 patients

		Mortality	
		Unadjusted	Adjusted^a^
		HR (95%CI)	HR (95%CI)
Indigenous status	Indigenous (vs. non-Indigenous)	1.16 (1.06–1.27)	1.08 (0.96–1.20)
Socio-demographic factors			
Age group (years)	50 years and over (vs. 20–49 years)	1.69 (1.58–1.80)	1.57 (1.45–1.69)
Gender	Male (vs. Female)	1.20 (1.14–1.26)	1.06 (1.00–1.12)
Marital status	No partner (vs. Married/De Facto)	1.12 (1.07–1.18)	1.07 (1.01–1.13)
Rurality of residence	Outside major city (vs. Major city)	1.17 (1.12–1.23)	1.13 (1.07–1.20)
Socioeconomic advantage and disadvantage	Q4, Q5 (vs. Q1, Q2, Q3)	1.16 (1.11–1.22)	1.04 (0.98–1.10)
Presumed aetiology			
Alcohol		1.33 (1.27–1.40)	1.06 (0.99–1.14)
Cryptogenic/NALFD/NASH		0.87 (0.82–0.91)	0.93 (0.87–0.99)
Chronic HCV/HBV		0.92 (0.86–0.97)	0.89 (0.83–0.95)
Comorbidities at index admission			
Charlson comorbidity index	> 0 (at least one comorbidity) vs. 0 (none)	2.27 (2.16–2.39)	1.46 (1.36–1.56)
Diabetes		1.40 (1.33–1.49)	n/s
Cancer		2.79 (2.62–2.96)	2.59 (2.26–2.97)
Complications of cirrhosis at index admission			
Ascites		1.81 (1.72–1.90)	1.47 (1.37–1.57)
Gastrointestinal bleeding		0.72 (0.68–0.76)	n/s
Hepatocellular carcinoma		2.42 (2.26–2.58)	0.93 (0.80–1.08)
Health service use			
Pharmacist		1.56 (1.46–1.66)	1.08 (1.00–1.16
Dietician		1.64 (1.56–1.73)	0.95 (0.88–1.02)
Social worker		1.95 (1.84–2.06)	1.28 (1.19–1.38)
Occupational therapist		1.93 (1.80–2.04)	n/s
Physiotherapist		1.96 (1.86–2.06)	1.14 (1.07–1.23)
Referral source	Emergency department presentation vs. not	1.78 (1.69–1.87)	1.56 (1.46–1.67)

Hazard ratio (HR); ^a^ HRs adjusted for length of stay, year of index admission, and all variables in the table except those marked n/s (variable not selected as a predictor)

As Indigenous Australians are overrepresented in rural/remote and socio-disadvantaged areas [24], we examined if there was interaction between Indigenous status and place of residence in relation to survival. There was no evidence of interaction between Indigenous status and rurality of residence (*p* = 0.17) or between Indigenous status and residence in the ‘most disadvantaged’ area (*p* = 0.27).

## Discussion

Cirrhosis was the 17th leading cause of death globally during 1990–2010 [25], the 22nd leading cause of death in Australia in 2017, and 6th leading cause of death for Indigenous Australians in 2017 [26]. There are systematic differences between the death rates for leading causes in Australia, which are consistently higher in Indigenous Australians. The largest differences are seen for deaths from diabetes (e.g. 5.2 times higher for Indigenous vs. non-Indigenous Australians) and cirrhosis and other diseases of the liver (3.7 times higher for Indigenous vs. non-Indigenous Australians). Understanding the clinical and epidemiological differences in hospital presentation and survival for Indigenous Australians with cirrhosis is critical if we are to improve programs of care for liver disease and address the mortality gap. Indigenous Australians admitted to hospital with cirrhosis are significantly younger, have more emergency presentations, longer hospital stay, more frequent readmissions occurring with greater comorbidity, more alcohol-related cirrhosis, and poorer survival than their non-Indigenous counterparts. The higher proportion of emergency presentation and alcohol-related cirrhosis among Indigenous patients were important factors explaining the survival deficit.

Late presentation with liver disease with advanced complications such as ascites is associated with poorer prognosis [27, 28]. In this study, the presence of ascites at the index admission was associated with poorer survival. A striking difference between Indigenous and non-Indigenous patients was seen for the source of admission and length of hospital stay. For over two-thirds of Indigenous Australians the referral source for their hospital admission was an emergency presentation, whereas this route accounted for less than half of admissions for non-Indigenous Australians. In addition, Indigenous patients had fewer 1-day admissions than non-Indigenous Australians. One-day admissions are likely to have a different clinical significance from that of longer admissions and usually represent delivery of planned care for diagnostic or therapeutic procedures. These findings suggest that many Indigenous Australians with cirrhosis may not be receiving a coordinated model of care for cirrhosis such as action plans for dealing with cirrhosis complications or access to day procedure units for planned management of ascites.

The current study showed that for individuals with cirrhosis who are Indigenous, alcohol was a significant contributing factor to the poorer survival. In 2015, alcohol was the sixth highest risk factor contributing to the burden of disease in Australia [29]. In 2016, Indigenous Australians were more likely to abstain from drinking alcohol than non-Indigenous Australians (31% vs. 23%, respectively), and this has been increasing since 2010. However, among those who did drink, a higher proportion of Indigenous Australians drank at risky levels compared to non-Indigenous Australians (35% of Indigenous Australians aged 14+ exceeded ‘single occasion of drinking’ risk guidelines vs. 25%) [30]. The ongoing legacy of European colonisation, dispossession, social disruption and intergenerational trauma [31, 32] have led to a multitude of problems including alcohol misuse and poor mental health among Indigenous Australians. Substance abuse, including alcohol, is a common way for victims of intergenerational trauma to cope with their symptoms as it offers a ‘quick fix’ to feelings of anxiety and despair [32]. A study conducted in Queensland and New South Wales has shown that while the rate of common mental disorders (e.g. mood, anxiety, and substance use including alcohol) among Indigenous Australians was up to 6.9 times higher than those of the Australian population [33], for Indigenous Australians living on traditional lands and in remote areas the rate of common mental disorders was half compared to those living in major cities or regional areas [33]. This suggests that connection to their traditional lands and communities is an important determinant of their mental health [33]. Although there has been some success in the reduction of harmful alcohol consumption among Indigenous communities [30], more needs to be done to prevent and minimise alcohol-related harm among individuals who consume alcohol. Intervention programs that pay attention to culture, tradition, and connection to homeland/traditional country may help decrease alcohol misuse among Indigenous Australians.

It is recognised that there is a high burden of comorbidity in patients with cirrhosis [34]. Moreover, the prevalence of overweight and obesity among the Indigenous adult population is higher across all age groups [2] placing them at greater risk of cardiometabolic conditions and fatty liver disease. In this study, despite being a significantly younger population, there was a higher prevalence of diabetes, chronic renal disease and congestive heart failure among Indigenous compared to non-Indigenous Australians. Indigenous people with cirrhosis required longer hospital stays and had more frequent readmissions. Addressing this mortality and morbidity gap requires greater integration between primary care, Aboriginal Community Controlled Health Organizations and liver specialists, so that greater opportunities are found to optimise community care and focus on prevention and secondary prevention post-discharge. Optimal care of patients with decompensated cirrhosis is often complex requiring a multidisciplinary approach [15]. The high burden of comorbidity coupled with the higher proportion of emergency presentations among Indigenous patients may explain the longer hospital stay compared with their non-Indigenous counterparts. These data highlight the importance of multidisciplinary management to address their complex health care needs and ensure optimal coordination of care. Moreover, it is important to consider the complex interplay of socio-environmental factors, [2] racism [3], and lack of adequate access to health care that contribute to the high burden of comorbidity and obesity in Indigenous patients.

The patient’s age is a strong predictor of mortality [7, 28], as older age leads to more advanced disease and increased risk of comorbid conditions which contribute to poorer patient outcomes. As Indigenous patients presented at younger age than non-Indigenous patients, univariable analysis of overall survival was confounded by age-group. When adjusted for age-group in multivariable analysis, the hazard ratio increased by 13%.

In this study, residence outside of a major city was associated with poorer survival. Australia is a vast country with an universal health-care system and people living outside major cities commonly have less access to health services and higher rates of hospitalisation and mortality [35]. In particular, hepatology services are largely limited to tertiary and large regional hospitals, and consequently many Indigenous Australians with cirrhosis living in rural areas may have to travel long distances or have limited access to hepatology services. There is a need to embrace other models of care to service rural and remote areas more effectively. Chronic care models that provide patient-centred care and improve communication and information transfer between hospital-based specialists and primary care clinicians (such as use of Liver Nurse Coordinators, structured co-management plans, the ECHO Model™) may help improve survival of patients living outside major cities [15].

Key strengths of this study included the use of population-based data and use of widely accepted and validated coding algorithms for cirrhosis, comorbidities, and identification of Indigenous status from hospital linked data [7, 17, 21]. Therefore, our findings may be generalizable to the broader population of Indigenous Australians admitted to hospital with cirrhosis. Code lists for identification of patients with cirrhosis, cirrhosis-related complications, and aetiology were confirmed by hepatologists [7]. However, the potential misclassification of presumed aetiology, co-factors and comorbidities is a potential limitation. It is possible that patients included in this study had additional comorbidity that was not relevant to their hospitalization and therefore not recorded.

## Conclusion

Our data suggests that despite greater health care service utilization, Indigenous patients with cirrhosis may benefit from greater care coordination to reduce emergency presentations and increase access to planned (likely shorter) episodes of care. The increased prevalence of emergency presentation among Indigenous patients suggests missed early opportunities for early intervention to prevent progressive complications and hospital readmissions e.g. supporting efforts to reduce alcohol consumption in those with alcohol use disorder, attention to dietary restrictions. Reducing the mortality gap requires greater integration between primary care, community care organizations and liver specialists to intervene in these disease processes before cirrhosis has resulted, and to prevent subsequent decompensation events. Further research, including qualitative data, is needed to better understand potential limitations and opportunities to better bring Indigenous Australians with cirrhosis to care, and develop culturally appropriate targeted interventions for Indigenous Australians with cirrhosis.

## Data Availability

Due to privacy constrains associated with our ethics approval, the datasets used during the current study are not publicly available.

## Abbreviations

Charlson index: Charlson Comorbidity Index
CI: Confidence interval
HBV: Hepatitis B virus
HCC: Hepatocellular carcinoma
HCV: Hepatitis C virus
HR: Hazard ratio
IQR: Interquartile range
NAFLD: Non-alcoholic fatty liver disease
NASH: Non-alcoholic steatohepatitis
T2D: Type 2 diabetes
